# Critical appraisal of an article on factitious schizophrenia

**DOI:** 10.4103/0019-5545.31609

**Published:** 2006

**Authors:** Rajnish Raj, Balwant Singh

**Affiliations:** * Senior Resident Department of Psychiatry, GMC and Rajindra Hospital, Patiala, Punjab; ** Associate Professor Department of Psychiatry, GMC and Rajindra Hospital, Patiala, Punjab

Sir,

The study by Grover *et al.*^1^ attempts to delineate a case report of factitious psychological symptoms suggestive of schizo-phrenia with empirical evidence in the literature regarding the existence of these types of cases.^2^ There is nothing unique about it; rather, Roger *et al.*^3^ questioned its diagnostic legitimacy in earlier studies. It highlights the diagnostic dilemma faced by professionals in recognizing the disease as a whole rather than in part. The attempt was like unravelling the road less travelled on borderline personality disorder (BPD) by the Indian psychiatric fraternity. The following points need reappraisal: Was it actually a factitious disorder, true or feigned insanity? Then why was Gestalt not attempted before making a diagnosis of borderline personality disorder? The pheno-menological construct in the case report is fraught with errors.

The age of onset of illness, diagnosis and duration of treatment are not properly delineated. The patient reported a transient attack of blindness that lasted for 12 hours. Besides ophthalmology, the neurologist's opinion in view of the differential diagnosis of occipital seizure, late age onset Gastaut with occipital spikes or basilar migraine was not sought. At the age of 17, the patient developed a seizure and attempted suicide when he was 18. Was it due to an emotionally unstable personality disorder (impulsive type)? Again, in the family history, the authors did not take into consideration the interaction of the patient within the family, level of bonding and attachment, handling of stress, and genetic load for psychiatric vulnerability. Regarding social and interpersonal relationships, these were stated to be normal. How is it possible that a person who was diagnosed according to the IPDE,^4^ WHO as having an enduring unstable personality disorder (impulsive type) had normal social and interpersonal relationships? The authors have not elaborated the reason for the decline in academic performance. In the personal history, there was no mention of drug history or substance abuse, peer group relationship and sexual history. The findings of the mental status examination and general physical examination are not mentioned in the report. It was cited that initially the patient had not intended to feign the psychosis but later when his father started giving him due care, he kept on maintaining the illness to save his mother. The authors have tried to put forth a hypothesis of Munchausen syndrome. According to Bursten (as cited by Cheng and Hummel),^5^ the major features of the syndrome are dramatic presentation, pseudologia fantastica and wandering. The case does not fulfil the criteria for factitious disorder due to unintentional production of symptoms, motivation to assume a sick role and presence of secondary gain. Hay^6^ concluded that simulation schizophrenia is a prodromal phase of psychosis occurring in extremely deviant premorbid personalities. Gunderson *et al.*^7^ concluded that in factitious psychosis, the clinician should suspect the possibility of BPD in which the patient develops psychosis under stressful conditions. Certainly, the intense instability and frequent manipulativeness of borderline patients calls into question the soundness of factitious disorders in the presence of such disorder. It exemplifies the complexity of diagnosing dis-simulation in psychiatric patients and recommends further study.
